# Single-Nucleus Transcriptional Profiling Revealed Cell Diversity and Albino Mutation Mechanism in the Skin of *Channa argus*

**DOI:** 10.3390/ijms27021023

**Published:** 2026-01-20

**Authors:** Lu Zhang, Jian Zhou, Qiang Li, Hongyu Ke, Zhipeng Huang, Zhongmeng Zhao, Han Zhao, Chengyan Mou, Wei Fan, Yuanliang Duan

**Affiliations:** 1 Fisheries Research Institute, Sichuan Academy of Agricultural Sciences (Sichuan Fisheries Research Institute), Chengdu 611731, China; zhanglu425@163.com (L.Z.); zhoujian980@126.com (J.Z.); liq7920@126.com (Q.L.); m13658000227@163.com (H.K.); h3392078@163.com (Z.H.); 18227552594@163.com (Z.Z.); zhaohan232323@163.com (H.Z.); 15927383463@163.com (C.M.); 2 Neijiang Academy of Agricultural Sciences, Neijiang 641000, China

**Keywords:** *Channa argus*, snRNA-seq, pigment cells, skin

## Abstract

Body color is the most prominent phenotypic trait in animals. To investigate the molecular regulatory mechanisms underlying skin pigmentation and body color in *Channa argus*, single-nucleus RNA sequencing technology was employed to analyze cell diversity and functional changes in the skin of normal and albino *C. argus*. Three pigment-related cell types, seven immune-related cell types, and nine other skin-related structural and functional cell types were identified. The skin of albino *C. argus*, which appears white to the naked eye, contains numerous melanocytes and iridophores with reflective silver properties. Compared to normal *C. argus*, melanocytes in albino individuals contained fewer melanin granules, while iridophores exhibited increased chromogenic substances. Melanocyte-specific genes—*kitlg*, *myo5a*, and *scarb1*—were significantly downregulated in albino melanocytes (*p* < 0.05). Conversely, iridophore-specific genes *alk*, *pnp*, and *gpnmb* were significantly upregulated in albino skin, whereas *mlph* was significantly downregulated (*p* < 0.05). Weighted gene co-expression network analysis revealed that *scarb1* was associated with the melanocyte module, *alk* was identified as a core gene, and *pnp* was linked to the iridophore module. Functionally, *scarb1* is involved in pigment transport, *pnp* in purine synthesis, and *alk* is essential for iridophore development. Therefore, *scarb1*, *pnp*, and *alk* may be correlated to albinism in *C. argus*. Overall, this study constructed a single-cell transcriptional atlas of *C. argus* skin, providing valuable reference data for further research into the regulatory mechanisms governing body color formation and maintenance in this species.

## 1. Introduction

*Channa argus* is the most widely distributed and productive species in the Channidae [1], commonly known as the northern snakehead. In 2024, the production of *C. argus* reached 595,498 tons, establishing it as a significant commercial fish species in China [2]. Two main variants of *C. argus* are cultured in China: the normal *C. argus*, which displays a decorative pattern of alternating black and white colors, and the albino *C. argus*, an albino variant characterized by a white or incanus body without any black patches [3,4,5]. The albino *C. argus* is highly valued by consumers for its delicious taste, medicinal properties, and ornamental appeal [5]. The white body color is a defining trait of the albino *C. argus* strain “Yulong No. 1” (new aquatic species registration number: GS-01-005-2022). This white coloration is stably inherited by offspring; however, the molecular regulatory mechanisms underlying the albino mutation in *C. argus* remain incompletely understood.

Albino mutation is a color variation widely observed in nature, ranging from coelenterates to mammals [6]. This mutation has been reported in numerous fish species, including rainbow trout, yellow catfish, and cavefish [7,8,9]. Some studies suggest that albinism in animals results from physiological, environmental, or genetic factors that disrupt the development and differentiation of melanocytes or melanin synthesis [10,11]. Although various factors influence body color expression in animals, the development and distribution of pigment cells—primarily determined by genetics—remain the main determinants of body color. The diversity of body colors and patterns in fish is governed by a complex genetic basis and regulatory mechanisms [12,13]. Molecular techniques have elucidated pigmentation mechanisms in some fish species; for example, the origin and types of pigmented cells in the skin and the mechanisms underlying stripe formation in zebrafish have been characterized [14,15,16]. Hundreds of genes regulate the differentiation and formation of pigment cells in fish [17,18,19]. Albino mutations in certain fish resemble those in mammals and are closely associated with mutations in the tyr gene. Mutations in tyr in zebrafish and half-smooth tongue sole result in an albino phenotype [20,21]. However, not all fish albinism is linked to tyr mutations. Research has demonstrated that mutations and altered expression of other single genes—such as *tyrp1*, *mc1r*, *mitf*, *dct*, *slc24a5*, *hps4*, and *oca2*—can cause varying degrees of melanin deficiency or albinism [22,23,24,25,26,27,28]. Nonetheless, the mechanisms underlying body color formation in fish are highly complex, and depigmentation may be influenced by one or multiple genes, with variations among species [29].

To investigate the albino mutation mechanism in *C. argus*, previous studies analyzed the average gene expression levels in the skin tissues of normal and albino *C. argus* using quantitative real-time polymerase chain reaction and traditional transcriptomic sequencing techniques [30,31,32]. However, the intercellular heterogeneity of gene expression within the skin tissues of these two *C. argus* variants has not been reported. Although some studies have examined the pigmentation characteristics of albino *C. argus* [30], the differences in skin cell composition between albino and normal *C. argus*, as well as whether gene expression within the same cell types is comparable, remain unknown. Single-nucleus RNA sequencing (snRNA-seq) technology enables high-throughput sequencing by amplifying trace amounts of whole transcripts from individual cells isolated from animal tissues, thereby elucidating gene–phenotype relationships at the single-cell level [33,34]. snRNA-seq has been widely applied In biology and medicine, advancing related research and applications [34], including the construction of the human cell landscape and the mapping of the mouse cell atlas at single-cell resolution. However, a comprehensive fish cell atlas has yet to be completed [35,36].

In this study, single-cell maps of normal and albino *C. argus* skin were constructed using 10× Genomics single-nucleus sequencing to elucidate the mechanisms underlying albinism in *C. argus*. Analyzing the cellular composition and gene expression profiles of differently pigmented skin will provide valuable reference data for constructing a comprehensive single-cell map of fish skin.

## 2. Results

### 2.1. Deconstructing C. argus Skin

**Figure 1:**
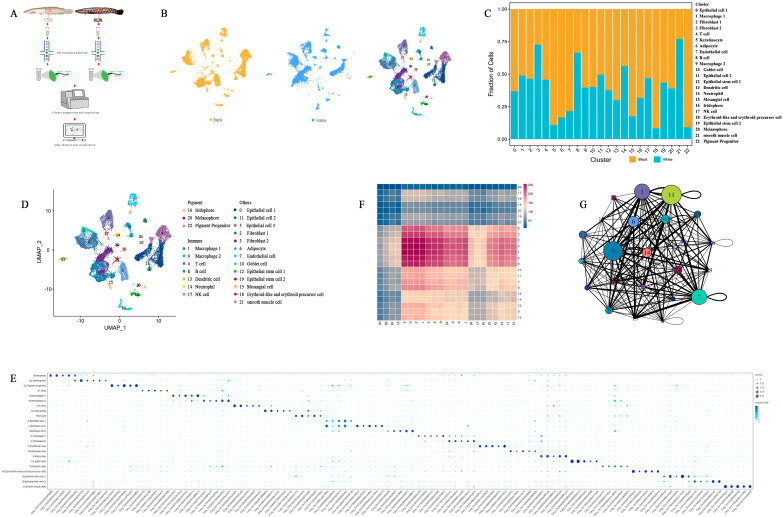
Deconstructing *Channa argus* skin. (**A**) The overall strategy for cell sorting and scRNA-seq analyses. (**B**) UMAP visualization showing all cell states found in normal and albino *C. argus* skin scRNA-seq dataset. Black: normal *C. argus* (black group), white: albino *C. argus* (white group). (**C**) Stack of the percentage of cells in skin samples of normal and albino *C. argus* in each cell cluster. (**D**) UMAP visualization displaying all cells separated into 23 subtypes. Each dot represents a cell, and cells belonging to different cell subgroups are colored differently. (**E**) Dot plot showing the expression of the known cell-type marker genes and new candidate markers for each cell state in (**D**). (**F**) Ligand–receptor pairing heatmap. Horizontal is the ligand cell subgroup, and vertical is the receptor cell subgroup. The number of ligand–receptor pairs in each pair of cells is represented by different colors. The bluer the color, the fewer the ligand–receptor pairs in this pair of cells, and the redder the color, the greater the number of ligand–receptor pairs in this pair of cells. (**G**) Diagram of the cell interaction network. Bubble size was determined by the number of significantly enriched receptor pairs between the subgroup and all interacting clusters. The larger the bubble size, the larger the total number, indicating a stronger subgroup association in the population. The line represents the number of significantly enriched receptor pairs between the clusters, and the thickness of the line is determined by the number of significantly enriched receptor pairs between the clusters. The thicker the line, the greater the number of significantly enriched receptor pairs between clusters, and the stronger the communication relationship between the clusters.

To gain a deeper understanding of the cell types in *C. argus* skin and the molecular regulatory mechanisms underlying the albino mutation in *C. argus*, the skins of normal and albino *C. argus* were extensively profiled by scRNA-seq. The overall strategy for cell sorting and scRNA-seq analysis is illustrated in Figure 1A. A total of 16,410 skin cells passed quality control, with 9586 cells obtained from normal *C. argus* and 6824 cells from albino *C. argus*. Cell types from all samples were classified together based on UMAP visualization for dimensionality reduction and unsupervised clustering. DEGs were identified to annotate the cell clusters, resulting in the identification of 23 cell clusters present in both normal and albino *C. argus*. The UMAP visualization is shown in Figure 1B. The number and proportion of different cell clusters in albino *C. argus* changed significantly compared to those in normal *C. argus* (Figure 1C).

Comparing gene expression between normal and albino *C. argus*, the specific upregulated genes in each cell cluster were similar across cell subpopulations. Cell annotation was performed using SingleR, which initially identified cell types based on a skin marker gene database from human and mouse (Figure S1). Subsequently, using reference cell types, the main cell types in the skin of *C. argus* were re-identified by marker genes within each cluster (Figure 1E and Table S1). The genes *Sox10*, *Kitlg*, *Cpeb2*, *Pax7*, and *Scarb1* are specifically expressed in cluster 20, among which *Sox10*, *Kitlg*, and *Cpeb2* have been identified as specifically expressed in melanophores [37,38,39]. The genes *Gpnmb*, *Alk*, *Pnp*, *Prtfdc1*, and *Myo5a* are specifically expressed in cluster 16, with *Gpnmb* and *Alk* further identified as being specifically expressed in zebrafish iridophores [40,41]. The genes *Mbl*, *Pdia2*, *Pou2f3*, *Slc22a2*, and *Tfap2a* are specifically expressed in cluster 22, with *Slc22a2* and *Tfap2a* further identified as being specifically expressed in zebrafish pigment progenitors [18]. Three pigment-related cell clusters were identified: melanocytes, iridophores, and pigment progenitors (Figure 1D,E). Based on cell marker genes in the PanglaoDB and CellMarker databases, seven immune-related cell clusters were identified, including two macrophage subtypes, B cells, T cells, natural killer (NK) cells, neutrophils, and dendritic cells. The skin of *C. argus* contained a large number of immune-related cells (Figure 1D,E). Additionally, nine other clusters of skin-related structural and functional cell types were identified, including epithelial cells, fibroblasts, endothelial cells, mesangial cells, adipocytes, goblet cells, erythroid-like and erythroid precursor cells, epithelial stem cells, and smooth muscle cells (Figure 1D,E).

Cell communication offers new research avenues to elucidate specific cellular behaviors. The number of ligand–receptor pairs expressed by each cell pair was determined through an analysis of ligand–receptor pair abundance (Figure 1F). An interactive network diagram was constructed to illustrate the communication relationships between cells (Figure 1G). The results revealed that cluster 0 (epithelial cells 1), cluster 11 (epithelial cells 2), cluster 1 (macrophages), and cluster 7 (endothelial cells) occupied central positions in the cell communication network, with these cell subgroups exhibiting strong correlations with other cell clusters (Figure 1G).

### 2.2. WGCNA

**Figure 2:**
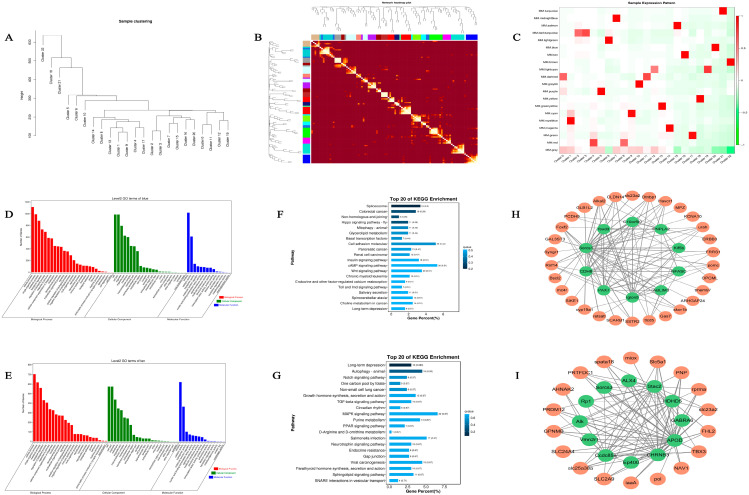
WGCNA. (**A**) Hierarchical clustering of all cell subpopulations. All cell subpopulations were hierarchically clustered according to the expression levels of all genes, and each cell subpopulation was regarded as a cluster. The closer the relationship between the cell subpopulations, the closer the distance. (**B**) Module–gene correlation heatmap. Each color block of the coordinate axis represents a module. Correlation analysis of the genes contained in the modules. Each row and column represents a gene, and the darker the color of each point (white → yellow → red), the stronger the connectivity between the two genes corresponding to the row and column (stronger Pearson’s correlation). (**C**) Sample expression pattern heatmap. The abscissa is the cell subgroup, and the ordinate is the module; red represents a high expression level, and green represents a low expression level. (**D**) Melanocyte and (**E**) iridophore histograms of the GO enrichment sorting. The abscissa indicates the second-level GO term, and the ordinate indicates the number of genes in the term. (**F**) Melanocyte and (**G**) iridophore KO enrichment bar graphs. The top 20 pathways with the smallest *p*-value were used to draw the graph. The ordinate indicates the pathway, and the abscissa indicates the percentage of the number of pathways in all differences. The darker the color, the smaller the *p*-value. The value in the column indicates the number of pathways and the *p*-value. (**H**) Melanocyte and (**I**) iridophore network diagram of top 100 relationship module connectivity pairs; green circles represent genes at the core position, and orange circles represent genes associated with core genes.

Hierarchical clustering was performed on all cell subpopulations based on the expression of all genes, with each cell subpopulation treated as a separate cluster. The hierarchical clustering of skin cells from the two distinct color morphs of *C. argus* is shown in Figure 2A. The clustering results for all cell subpopulations were consistent with those for the cell types (Figure 1D), with subpopulations sharing similar functions clustering together. Twenty gene network modules were identified from the WGCNA of the scRNA-seq data (Figure 2B). Subsequent expression pattern analysis revealed modules significantly associated with specific cell clusters.

The pigment cell modules were used to extrapolate the hub-gene networks; the tan module was most closely associated with cluster 16 (iridophores), while the blue module was most closely associated with cluster 20 (melanocytes) (Figure 2C). The genes in the tan module (954 genes) and the blue module (1555 genes) were analyzed by GO. The gene expression profiles of the two modules were similar; major GO terms in both the tan and blue modules were related to the regulation of biological processes, including cellular processes, metabolic processes, and biological regulation (Figure 2D,E). Moreover, genes in both modules were enriched in many of the same GO terms, such as cells, cell parts, organelles, cellular components, binding, melanocytes and iridophores, and catalytic activity. However, the KO enrichment analysis revealed that the genes in the two modules were enriched in distinct signaling pathways: genes in the blue module were mainly involved in the Wnt signaling pathway, whereas genes in the tan module were primarily involved in the mitogen-activated protein kinase (MAPK) signaling pathway and purine metabolism (Figure 2F,G). To further explore the key genes in the tan and blue modules, network diagrams of the top 100 connectivity pairs were inferred for each module. 10 core genes in the blue module (*pax7*, *cdh8*, *sorcs1*, *foxd3*, *c10orf90*, *pnpla2*, *kif5b*, *nfasc*, *ablim3*, and *iglon5*) (Figure 2H, green circles) and 30 associated genes (e.g., *scarb1*, *slc23a2*, and *mc4r*) (Figure 2H, orange circles) were identified. In the tan module, 12 core genes (*alk*, *rp1*, *sorcs3*, *alx4*, *stac2*, *hdhd5*, *gabra6*, *apod*, *chrnb3*, *ep400*, *ccdc85a*, and *vmn2r1*) (Figure 2I, green circles) and 18 associated genes (e.g., *pnp*, *gpnmb*, *slc2a9*, *scl23a2*, and *scl5a1*) (Figure 2I, orange circles) were identified.

### 2.3. Gene Expression in Pigment Cells

**Figure 3:**
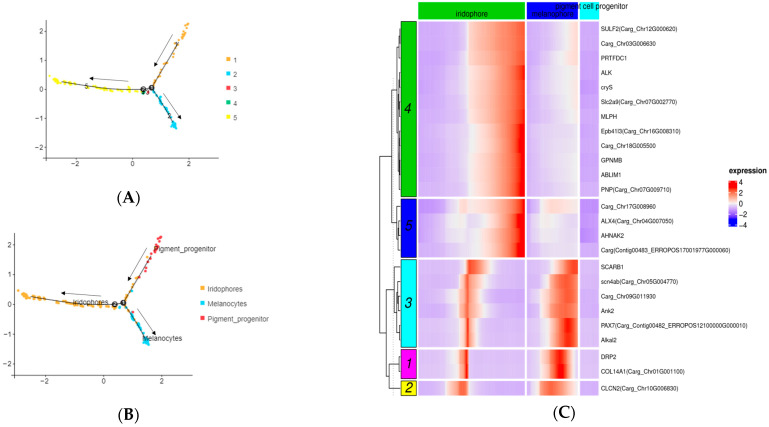
Pseudotime analysis. (**A**) Pseudotime analysis of the pigment cell differentiation states diagrams. The distribution of cells in different differentiation states in the cell track; different colors represent different differentiation states. (**B**) Pseudotime analysis of the pigment cell differentiation trajectory diagrams. (**C**) BEAM revealed dynamics of gene expression over pseudotime for each pigment cell branch. The abscissa represents the pseudotime axis, and the ordinate represents the gene. The legend value represents gene expression. The larger the value, the higher the expression of the gene at the pseudotime point.

The pigment cell lineage and the pseudotime differentiation trajectory of the pigment cell lineage were constructed using the Monocle algorithm to characterize transcriptional dynamics during lineage maturation. Cells were ordered pseudotemporally, revealing a differentiation trajectory in which two pigment cell types arise from a common progenitor. Five cellular states were identified based on three pigment-correlated cell clusters (Figure 3A), and the pigment cell differentiation trajectory is shown in Figure 3B. BEAM revealed gene expression dynamics over pseudotime for each pigment cell branch. Nine genes, such as *scarb1* and *pax7*, were upregulated early in iridophores but expressed later in melanocytes. Additionally, sixteen genes, such as *alk*, *mlph*, *gpnmb*, and *pnp*, were specifically upregulated later in iridophores, as confirmed by BEAM analysis (Figure 3C). These genes may influence pigment cell differentiation fate and regulate pigment cell development through their orderly expression.

**Figure 4:**
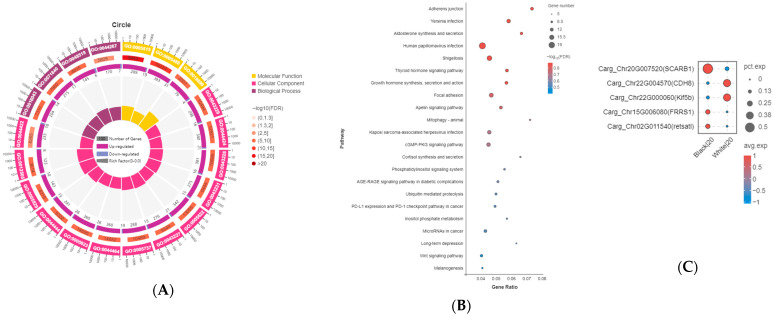
DEGs analysis of cell cluster 20 in the two distinct color morphs of *Channa argus*. Note: (**A**) GO enrichment circle diagram of the DEGs. First circle: top 20 GO terms enriched; outside the circle is the coordinate ruler of the number of genes. Different colors represent different ontologies. Second circle: number of GO terms in the background gene and the Q value. More genes are shown by a longer bar. Smaller Q values have a redder color. Third circle: bar graph of the ratio of upregulated genes, where dark purple represents the ratio of upregulated genes. Fourth circle: Rich factor value of each GO term. (**B**) KEGG enrichment bubble diagram of the DEGs. Top 20 enriched pathways, including the Wnt signaling pathway and melanogenesis pathway; the larger the Rich factor, the higher the degree of enrichment. (**C**) Dot plot showing some of the DEGs in the melanocyte subsets between albino and normal *C. argus*.

A total of 552 DEGs were identified in the melanocytes of *C. argus*. Among these, the expression of 510 genes was significantly upregulated, while 42 genes were significantly downregulated in the skin of albino *C. argus* compared to normal *C. argus* (*p* < 0.05). To explore the functional characteristics of the cellular pathways involved from a macroscopic perspective, GO annotation and KO functional analyses were performed on the upregulated and downregulated genes. The GO annotation revealed that the melanocyte cluster contained DEGs enriched in molecular functions such as protein binding (GO: 0005515), binding (GO: 0005488), and enzyme binding (GO: 0005488) (Figure 2B). Additionally, these DEGs were enriched in various cellular components, such as intracellular organelles (GO: 0043229) and intracellular parts (GO: 0044424) (Figure 4A). The KO functional analysis indicated that some DEGs in the melanocyte subsets were enriched in the thyroid hormone signaling pathway (ko04919), the Wnt signaling pathway (ko04310), and melanogenesis (ko04916) (Figure 4B). Based on the results of WGCNA, the differential expression of hub genes in melanocytes of normal and albino *C. argus* was further analyzed. The genes *cdh8* and *kif5b* were upregulated, while *scarb1*, *frrs1*, and *retsatl* were downregulated in albino *C. argus* among the hub genes in the blue module (*p* < 0.05) (Figure 4C). Among these differentially expressed hub genes, only *scarb1* may influence the differentiation fate of melanocytes, based on the results of pseudotime analysis.

**Figure 5:**
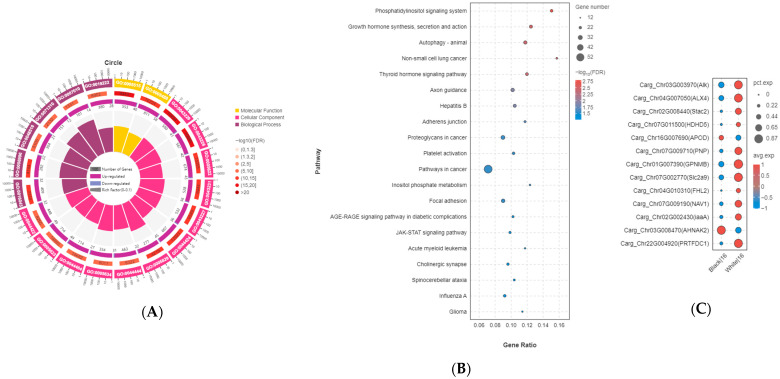
DEGs analysis of cell cluster 16 in the two distinct color morphs of *Channa argus*. Note: (**A**) GO enrichment circle diagram of the DEGs. First circle: top 20 GO terms enriched; outside the circle is the coordinate ruler of the number of genes. Different colors represent different ontologies. Second circle: number of GO terms in the background gene and the Q value. More genes are shown by a longer bar. Smaller Q values have a redder color. Third circle: bar graph of the ratio of upregulated genes, where dark purple represents the ratio of upregulated genes. Fourth circle: Rich factor value of each GO term. (**B**) KEGG enrichment bubble diagram of DEGs. Top 20 enriched pathways; the larger the Richfactor, the higher the degree of enrichment. (**C**) Dot plot showing some of the DEGs in the iridophore subsets between albino and normal *C. argus*.

A total of 1167 DEGs were identified in the iridophores of *C. argus*. Among these, the expression of 1087 genes was significantly upregulated, while 80 genes were significantly downregulated in the skin of albino *C. argus* compared to normal *C. argus* (*p* < 0.05). Several identical GO terms appeared in the top 20 GO terms for both iridophores and melanocytes, such as protein binding (GO: 0005515), binding (GO: 0005488), molecular functions and intracellular organelles (GO: 0043229), intracellular parts (GO: 0044424), and cellular components (Figure 5A). The KO functional analysis revealed that the DEGs in the iridophore subsets were primarily enriched in the thyroid hormone signaling pathway (ko04919) and cancer-related pathways (ko05200) (Figure 5B). Based on WGCNA, the differential expression of hub genes was further analyzed in iridophores of normal and albino *C. argus*. The genes *alk*, *pnp*, *gpnmb*, *alx4*, *stac2*, *hdhd5*, *slc2a9*, *fhl2*, *nav*, *iaaa*, and *prtfdc1* were upregulated, whereas *apod* and *ahnak2* were downregulated in albino *C. argus* among the hub genes in the tan module (*p* < 0.05) (Figure 5C). Among these differentially expressed hub genes, *alk*, *gpnmb*, *pnp*, *alx4*, *prtfdc1*, *slc2a9*, and *ahnak2* may influence the differentiation fate of iridophores based on pseudotime analysis results.

### 2.4. Morphology and Distribution of Pigment Cells

**Figure 6:**
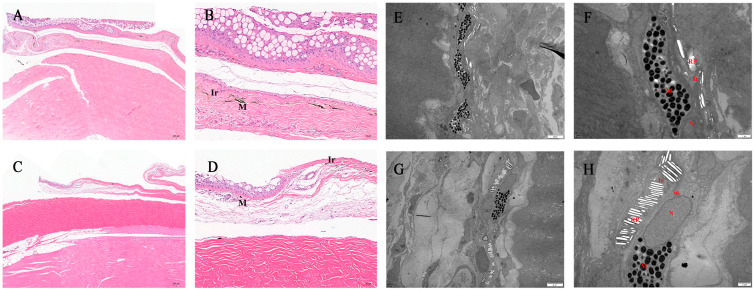
Tissue slices of *Channa argus* skin. Note: (**A**–**D**) H&E-stained images of the skin of the two color morphs of *C. argus* (n = 3). H&E-stained image of the skin of normal *C. argus* fixed directly after being cut on a dermatome; scale bars represent 200 μm (**A**) and 50 μm (**B**), respectively. H&E-stained image of the skin of albino *C. argus* fixed directly after being cut on a dermatome; scale bars represent 200 μm (**C**) and 50 μm (**D**), respectively. (**E**–**H**) Tissue slices of the skin samples under transmission electron microscopy (n = 3). The skin of normal *C. argus*; scale bars represent 5 μm (**E**) and 1 μm (**F**), respectively. The skin of albino *C. argus*; scale bars represent 5 μm (**G**) and 1 μm (**H**), respectively. M: Melanocyte, Ir: iridophore, N: nucleus, Mi: mitochondrion, RP: reflector plate.

The melanocytes appeared brown or black, and the iridophores were light gray after H&E staining (Figure 6A). Numerous melanocytes and iridophores were observed in the epidermis and loose dermis of normal *C. argus*, whereas only a few melanocytes and iridophores were detected in the skin of albino *C. argus*. The pigmented layer of the albino *C. argus* skin showed signs of degradation (Figure 6A–D). The number of pigment cells observed in skin tissue sections was consistent with the data obtained by scRNA-seq (Figure 1C). Melanocytes were examined by TEM at 12,000× magnification in skin sections of both normal and albino *C. argus*. The cell bodies were flat, nuclei were oval, heterochromatin was located near the nuclear membrane, and numerous melanin particles were present in the cytoplasm. At the same magnification, iridophores resembled melanocytes morphologically and were adjacent to them. No melanin granules were observed in the iridophore cytoplasm; however, various reflector plates were present (Figure 6B). In the normal *C. argus*, a few melanocytes were observed in the epidermis, while more melanocytes and some iridophores were found in the dermis. In contrast, only small numbers of melanocytes and iridophores were observed in the dermis of the albino *C. argus*. Melanocytes in the albino *C. argus* contained fewer melanin granules than those in the normal *C. argus*, whereas the number of reflector plates in the iridophores was higher than in the normal *C. argus* (Figure 6E–H).

## 3. Discussion

Body color is the most prominent phenotypic trait of animals, and the development and maintenance of fish body color involve precise and complex molecular regulation [13]. To investigate the mechanism of skin albinism, a study was conducted on the cellular characteristics and gene expression of various pigment cells in both normal and albino *C. argus* using scRNA-seq analysis. Three pigment-related cell types, seven immune-related cell types, and nine other skin-related structural and functional cell types were identified in the two color morphs of *C. argus*. The scRNA-seq data revealed novel markers and cell-type-specific expression patterns of some previously identified markers across all cell types.

The type, quantity, shape, size, and distribution of pigment cells, along with changes during different developmental stages, determine the diversity of fish body color and pattern [42]. This study focused on the type, quantity, and gene expression of pigment cells in the skin of two color morphs of *C. argus*. The types of pigment cells in the skin of albino *C. argus* did not differ from those in normal *C. argus*; however, the numbers of melanocytes and iridophores were significantly reduced, as shown by scRNA-seq results and tissue observations. TEM revealed that the skin of albino *C. argus* contained relatively fewer melanin granules within melanocytes and a greater number of reflective platelets in iridophores. Previous studies by Zhou et al. [33] microscopically observed melanocytes in the skin of albino *C. argus* and speculated that the irregular arrangement of melanocytes in the dermis and disrupted melanin synthesis might cause albinism. However, iridophores were neither observed nor analyzed in their study. In this study, the observations of melanocytes in both normal and albino *C. argus* skin are consistent with these earlier findings, as is the presence of a higher number of reflective platelets in the iridophores of albino skin compared to normal skin. Therefore, it is hypothesized that albinism in *C. argus* may be associated with a decrease in the number of pigment cells and melanin granules, alongside an increase in reflective platelets within iridophores.

Melanocytes are a type of pigment cell widely present in the dermis and epidermis of animals, and research on melanocytes is extensive. It has been confirmed that melanocytes are related to the development of albinism [43]. The melanin synthesis pathway is closely linked to pigmentation. Melanogenesis is regulated by multiple factors through complex pathways activated by hormonal, receptor-dependent, and receptor-independent mechanisms [44]. In this study, the DEGs in the skin melanocytes of normal and albino *C. argus* were annotated to the thyroid hormone signaling pathway, Wnt signaling pathway, and melanogenesis. The Wnt signaling pathway and melanogenesis have been shown to be associated with albinism in several fish species. For instance, albinism in the Oscar may be linked to the Wnt signaling pathway and melanogenesis [45], and albinism in yellow catfish and cichlids is also related to melanogenesis [8,46]. Wnt signaling plays a role in regulating ongoing aspects of melanocyte differentiation in zebrafish [47]. It functions in conjunction with *sox10* to drive *mitfa* expression during melanocyte fate specification [48]. Thyroid hormone regulates the adult striped pattern by limiting the expansion of neural crest-derived pigment cells. Additionally, thyroxine promotes terminal differentiation in melanocytes, ultimately limiting their total number in zebrafish [40]. Together, these melanocyte-enriched pathways likely play key roles in regulating melanocyte identity.

Unlike melanocytes, iridophores do not contain pigment particles; instead, their chromogenic substance is a crystalline form of guanine, which is highly reflective, as demonstrated in this study. Iridophores function like small mirrors that selectively reflect and absorb different colors. Previous studies have confirmed the absence of pigment particles in iridophores [49,50]. Additionally, iridophores can regulate the number of melanocytes during the formation of the adult pigment pattern in zebrafish [16,51]. Prior research has reported significant overlap in marker genes between zebrafish embryonic melanocytes and iridophores, suggesting that these cells may originate from a common precursor. Moreover, melanoblasts in zebrafish mutants can change their original cell lineage fate to become iridophores [52,53,54,55]. In this study, signature genes such as *myo5a*—an actin-based motor protein involved in melanosome trafficking in melanocytes—were co-expressed in both melanocytes and iridophores [56,57]. To understand the differentiation relationship between melanocytes and iridophores in *C. argus* and to identify key regulatory factors involved in pigment cell differentiation, the pseudotime analysis was performed, and the findings revealed that both pigment cell types originate from a common pigment progenitor, indicating a shared differentiation pathway. Furthermore, genes associated with differentiation fate in melanocytes and iridophores showed significant overlap. Genes upregulated during the middle stages of iridophore differentiation exhibited a similar expression pattern during the middle and late stages of melanocyte differentiation. However, genes upregulated in the late stages of iridophore differentiation did not show significant upregulation in melanocytes. Therefore, we speculate that early differentiated melanocytes in the skin of *C. argus* may have the potential to transform their original cell lineage fate and develop into iridophores. To further investigate gene expression characteristics in iridophores of the two color morphs of *C. argus*, DEGs in iridophores were primarily annotated to the thyroid hormone signaling pathway and cancer-related pathways. Notably, iridophore maturation in zebrafish is strongly influenced by thyroid hormone status [40]. The gene *alk*, which is essential for iridophore development and is involved in cancer-related pathways, was significantly upregulated in the skin of albino *C. argus* [41].

In this study, differentially expressed hub genes were identified: *scarb1* in melanocytes and *alk*, *gpnmb*, *pnp*, *alx4*, *prtfdc1*, *slc2a9*, and *ahnak2* in iridophores, which may influence the differentiation fate of pigment cells in the skin. Among these genes, *scarb1* is an important regulatory gene involved in carotenoid absorption and transport during fish pigmentation [58,59]. *Scarb1* is expressed in the intestinal tract of Atlantic salmon, where it facilitates carotenoid absorption, potentially contributing to the red flesh color of *Atlantic salmon* [60]. *Scarb1* may also be related to the formation of red fish skin, as it is expressed in red skin and fin stripes but is either not expressed or expressed at very low levels in the white skin and fin stripes of koi [61]. Some studies have reported that *scarb1* is involved in the deposition of dark spots on the body surface, with its expression level in the skin of koi with dark spots being higher than in red and white koi [62]. Additionally, research on human melanoma pigmentation has shown that *scarb1* expression is strongly correlated with the expression of mitf, a key gene in melanin deposition, and the melanin synthesis pathway [63]. This study suggests that the downregulation of the melanin-specific gene *scarb1* in the skin of albino *C. argus* may be a significant factor contributing to the degeneration of body color.

In iridophores, *alk*, *gpnmb*, *pnp*, *alx4*, *prtfdc1*, and *slc2a9* were upregulated, while *ahnak2* was downregulated in the albino *C. argus* in this study. *Alk* belongs to the receptor tyrosine kinase superfamily and is essential for the development of iridophores in zebrafish, mediated by the receptor tyrosine kinase ltk [41]. *Sox10*, *pax3*, and the downstream gene *alk* inhibit melanin synthesis and initiate the differentiation and expression of iridophores [64]. *Gpnmb* has been described as a component of the melanosome but is more highly expressed in iridophores than in melanocytes of zebrafish. *Gpnmb* has been suggested as a marker gene and may reflect platelet organelle biogenesis in iridophores [51]. The chromogenic substance in iridophores is crystalline guanine, and *pnp* plays a key role in the purine salvage pathway by catalyzing the conversion of guanosine to guanine [42,65]. *Prtfdc1* belongs to the purine/pyrimidine phosphoribosyltransferase family and has been identified as a genetic modifier of hypoxanthine phosphoribosyltransferase as well as a potential tumor suppressor [66,67]. In zebrafish, the guanine synthesis-related gene *prtfdc1* is significantly upregulated in iridophores compared to melanocytes [51]. In this study, skin sections of albino *C. argus* contained a greater number of reflector plates in the iridophores than those of normal *C. argus*. Together, these findings suggest that the upregulation of *alk* likely promotes the development of iridophores, while the upregulation of *gpnmb*, *pnp*, *alx4*, and *prtfdc1* likely promotes the formation of chromogenic substances in iridophores. Members of the *alx4* family are known to regulate the formation of skeletal elements in organisms; *alx4a* and *alx4b* are enriched in iridophores of zebrafish, but the role of *alx4* in regulating iridophore identity remains unclear [51,68]. *Slc2a9* has been suggested to function as a fructose and uric acid transporter and is likely associated with gout and leukemia in humans [69,70]. *Ahnak2* is a novel prognostic marker and correlates with immune infiltration in papillary thyroid cancer [71]. However, the functions of *slc2a9* and *ahnak2* in iridophores are not well understood.

However, no significant difference in the expression of *tyr*, a key gene involved in melanin synthesis [20], was detected in the skin of the two color morphs of *C. argus*. Previous studies on the mechanism of albinism have shown varying results. Zhou et al. [32] reported that the expression level of *tyr* in the skin of juvenile normal *C. argus* is higher than that in juvenile albino *C. argus*, based on qRT-PCR analysis. Conversely, Li et al. [30] conducted a transcriptomic study on the skin tissue of albino *C. argus* and concluded that the combined effects of missense mutations in the *hgd* and *adh5* genes, along with decreased expression of DEGs in the *tyr* metabolic pathway, were the primary causes of reduced melanosis in albino *C. argus*. The key genes *tyr* and *tyrp2*, which influence albinism in fishes, were not significantly differentially expressed between adult normal and albino *C. argus*. It is speculated that the expression levels of the *tyr* gene family, as key regulators of melanin synthesis, vary significantly during different growth stages of the fish, possibly due to the gradual transition of pigment patterns from juvenile to adult stages [30]. In this study, no significant differences were found in the expression levels of *tyr* and *tyrp2* in the pigment cells of the two color morphs of *C. argus*. Therefore, in conjunction with previous reports, it is speculated that *tyr* and *tyrp2* may not be the key genes affecting the production and maintenance of the albino phenotype in adult *C. argus*. Additionally, this study detected downregulated expression of *hgd* and *adh5* in the two color morphs, consistent with previous findings. It was also observed that *hgd* and *adh5* were relatively highly expressed in fibroblasts and relatively poorly expressed in pigment cells. Therefore, it is speculated that *hgd* and *adh5* may have functional roles in the fibroblasts of *C. argus*.

## 4. Materials and Methods

### 4.1. Ethics Statement

All animals collected for this study were handled in accordance with ethical guidelines. All fish handling and experimental procedures were approved by the Animal Care and Use Committee of the Fisheries Research Institute, Sichuan Academy of Agricultural Sciences (Sichuan Fisheries Research Institute), under approval number SYS-S20210016, approval date: 8 June 2021.

### 4.2. Experimental Animals and Sample Collection

The two color morphs of *C. argus* (black and white groups, weighing 1.0 ± 0.1 kg) were collected from the Neijiang aquaculture farm. Both the normal and albino groups were cultured in the circulating aquaculture system at the Fisheries Research Institute, Sichuan Academy of Agricultural Sciences (Sichuan Fisheries Research Institute), for 7 days under identical conditions. Three fish from each group were randomly selected. After anesthetization, the fish were wiped clean, and skin tissue was collected along the lateral line and washed with phosphate-buffered saline.

### 4.3. Tissue Slices

The skin samples were stored in a fixation solution for 48 h at 4 °C before being collected for paraffin embedding. Hematoxylin and eosin (H&E) staining was applied to the paraffin-embedded tissue blocks. Tissue dehydration, paraffin embedding, sectioning, and staining were performed by Lily Biotechnology Co. (Chengdu, China).

After fixation in 3% glutaraldehyde, the tissue was postfixed in 1% osmium tetroxide, dehydrated through a graded acetone series, and embedded in Epox 812. Methylene blue was used to stain semi-thin sections, while uranyl acetate and lead citrate were applied to stain ultrathin sections. The sections were examined using a JEM-1400FLASH transmission electron microscope (JEOL Ltd., Tokyo, Japan).

### 4.4. Cell Nuclear Extraction

Three pieces of skin tissue (approximately 1 cm^2^ each) were combined as a sample, chopped into 2 mm^3^ pieces at 4 °C, frozen, and stored in liquid nitrogen. The subsequent nuclear isolation experiment was initiated after confirming the RNA quality of the sample. Nuclear extraction was performed as previously described [72]. Briefly, following homogenization of the samples with a grinding rod, the homogenate was centrifuged, filtered through a 70 µm cell strainer, diluted with iodixanol, and centrifuged again (4 °C, 10,000× *g* for 20 min). After resuspension in nuclear cleaning solution, the nuclear suspension was filtered through a 40 µm filter and centrifuged (4 °C, 500× *g* for 5 min). Then, 100 µL of nuclear cleaning solution was added to resuspend the nuclear pellet. The nuclear suspension was assessed by trypan blue staining using a microscope and a hemocytometer (Biobase Kings Co., Jinan, China) to estimate the total number, concentration, and proportion of nuclei with intact nuclear membranes. The nuclear suspension concentration was adjusted to 700–1200 nuclei/µL for scRNA-seq analysis.

### 4.5. Sequencing and Data Quality Control

Gel Bead-In-Emulsions (GEMs) were used to partition thousands of nuclei, each labeled with a 10× barcode. Gene de novo cDNA libraries were prepared and sequenced, and the 10× barcodes were matched as individual reads to specific partitions comprising the standard Illumina paired-end constructs. The data were converted from raw BCL files to FASTQ files, and alignment and quantification of counts were performed using 10× Genomics Cell Ranger software (version 3.1.0) (Cell Ranger: http://support.10xgenomics.com/single-cell/software/overview/welcome, accessed on 15 January 2026). Briefly, reads with low-quality barcodes and UMIs were filtered out and mapped to the reference genome of *C. argus* (GenBank assembly accession: GCA_018997905.1). Reads uniquely mapped to the transcriptome and intersecting an exon/intron at least 50% were considered for UMI counting (Table S2). Prior to quantification, UMI sequences were corrected for sequencing errors, and valid barcodes were identified using EmptyDrops (1.22.0) [73].

### 4.6. Cell Clustering and Cell-Type Annotation

The R package Seurat (version 3.1.4; The R Foundation for Statistical Computing, Vienna, Austria) was used for dimensionality reduction, clustering, and analysis of the scRNA-seq data [74]. DoubletFinder (V2.0.4) [75] was employed to calculate the probability of multiplets in the GEMs (pANN value). The multiplet rate was determined by comparing the effective cell number (after Cell Ranger filtration) with the rate provided by 10× Genomics. Nuclei expressing fewer than 500 or more than 4000 genes, as well as nuclei with over 20% mitochondrial gene expression, were excluded. After removing low-quality nuclei, Harmony (V1.2.0) [76] was applied to integrate the data and correct for batch effects. First, principal component analysis (PCA) was performed on the combined dataset for dimensionality reduction. Harmony uses a soft k-means clustering algorithm to cluster the data in the reduced-dimensional space and probabilistically assigns nuclei to clusters to maximize dataset diversity within each cluster. Then, the global centroid of all datasets was calculated within each cluster, along with the centroid of each individual dataset. A correction factor for each dataset within each cluster was computed based on these centroids, and nuclei were adjusted to converge toward the global centroid. These steps were iterated until the clustering stabilized. Based on the cell subpopulation classification results, the Uniform Manifold Approximation and Projection (UMAP) method was used to visualize the single-nucleus subpopulation classifications.

Cell annotation was performed using SingleR (https://github.com/dviraran/SingleR, accessed on 15 January 2026) (SingleR 2.4.1.), which initially identified cell types by comparing the expression patterns of the nucleus to be classified with those of reference cell types. At present, the marker genes for various types of cells in fish are still not well established. Subsequently, the main cell types in the skin of the two groups were determined based on the marker gene populations within each group, using CellMarker (V2.0) [77], PanglaoDB (V1.5) [78], and other well-established marker genes from the literature.

### 4.7. Differential Expression Analysis

Using Wilcoxon rank-sum tests, the expression values of genes in each cluster were compared with those of all other nuclei [79]. Significant upregulation or downregulation was identified based on the following criteria: genes had to be overexpressed at least 1.28-fold in the target cluster and expressed in more than 25% of nuclei within that cluster, with a *p*-value ≤ 0.05. Gene Ontology (GO) terms are classified into different ontology types [80]. All peak-related genes were mapped to GO terms in the Gene Ontology database (http://www.geneontology.org/, accessed on 15 January 2026). The calculated *p*-values were corrected using the false discovery rate (FDR), with a threshold of ≤0.05. Differentially expressed genes (DEGs) with significantly enriched KEGG Orthology (KO) terms were identified based on terms in the KEGG database [81]. To identify pathways significantly enriched in DEGs, the FDR correction was applied to the calculated *p*-values, using a threshold of 0.05 [82].

### 4.8. Cell Communication Inference and Trajectory Analysis

CellphoneDB software (CellphoneDB V5) [83] was used to analyze the number of ligand–receptor pairs and their expression levels in cell pairs using a single-nucleus gene expression matrix. A cell interaction network diagram was then constructed to predict potential relationships between the clusters.

Using Monocle (Version 2.10.1), a matrix of nucleus and gene expression levels was analyzed to infer single-nucleus trajectories [84]. Monocle employs a pseudotime approach that sequences individual nuclei based on the expression patterns of key genes. By capturing the asynchronous biological processes within a single nucleus, Monocle positions the nucleus along a trajectory that corresponds to a biological process, such as cell differentiation, thereby simulating developmental changes over time. The dimensionality of the data is reduced to two dimensions, and nuclei are ordered with parameters sigma = 0.001, lambda = NULL, gamma = 10, and tolerance = 0.001 [85]. Cell differentiation sites were mapped using gene expression arrays generated by 10× Genomics. Trajectories representing different differentiation states, samples, and cell subpopulations are presented. Here, the term “state” refers to segments of the trajectory or branches created by Monocle. Key genes involved in development and differentiation were identified using an FDR threshold of less than 1 × 10^−5^. Additionally, the Monocle project developed Branch Expression Analysis Modeling (BEAM) to detect branch-dependent gene expression by comparing gene expression patterns across branches [86].

### 4.9. Weighted Gene Co-Expression Network Analysis (WGCNA)

Co-expression networks were constructed using the WGCNA (v1.47) package in R [87]. Gene expression values were imported into WGCNA, and the automatic network construction function was used to generate co-expression modules. By default, an unsigned TOM type was applied with a cut height of 0.7 and a minimum module size of 50. WGCNA assumes that gene networks follow a scale-free topology and defines a correlation matrix, gene expression similarity, and neighborhood functions to characterize gene networks. A hierarchical clustering tree based on the topological overlap of genes was then constructed. Different branches of the clustering tree represent distinct gene modules, where genes within the same module exhibit a higher degree of co-expression, while genes in different modules show lower co-expression. Finally, the associations between modules and specific phenotypes were investigated, and target genes related to phenotypes and gene networks were analyzed.

## 5. Conclusions

This study constructed a single-nucleus transcriptional map of *C. argus* skin and preliminarily identified genes that may be related to the formation and deposition of pigment cells in the skin of *C. argus*. This research provides important reference information for in-depth studies on the regulatory mechanisms underlying body color formation and maintenance in *C. argus*. However, previous studies have shown that the mechanisms of albinism in fish are not entirely consistent, making it a significant challenge to systematically elucidate the mechanism of albinism in *C. argus*.

## Data Availability

The original contributions presented in this study are included in the article/Supplementary Material. Further inquiries can be directed to the corresponding authors.

## Supplementary Materials

The following supporting information can be downloaded at https://www.mdpi.com/article/10.3390/ijms27021023/s1.
