# Supplementary material for: Single-Nucleus Transcriptional Profiling Revealed Cell Diversity and Albino Mutation Mechanism in the Skin of Channa argus
Source: Int J Mol Sci. 2026 Jan 20;27(2):1023. doi: 10.3390/ijms27021023 (PMC12841621; doi:10.3390/ijms27021023)
Supplement: Supplementary file 1 [file ijms-27-01023-s001.zip › ijms-4070565-supplementary.pdf]

Supplementary Materials

Table S1: Marker genes in cell clusters.

Cell type	GeneID	GeneName
Iridophore	Carg_Chr01G007390	GPNMB
Iridophore	Carg_Chr03G003970	Alk
Iridophore	Carg_Chr07G009710	PNP
Iridophore	Carg_Chr22G004920	PRTFDC1
Iridophore	Carg_Chr06G007200	Myo5a
Melanophore	Carg_Chr02G001820	SOX10
Melanophore	Carg_Chr05G003070	KITLG
Melanophore	Carg_Chr11G001950	CPEB2
Melanophore	Carg_Chr12G011580	PAX7
Melanophore	Carg_Chr20G007520	SCARB1
Pigment progenitor	Carg_Chr06G000430	MBL
Pigment progenitor	Carg_Chr08G003870	PDIA2
Pigment progenitor	Carg_Chr19G001410	Pou2f3
Pigment progenitor	Carg_Chr21G001860	SLC22A2
Pigment progenitor	Carg_Chr22G001600	TFAP2A
T cells	Carg_Chr01G002960	Cd6
T cells	Carg_Chr03G001630	LCK
T cells	Carg_Chr09G010140	SATB1
T cells	Carg_Chr23G001520	skap1
T cells	Carg_Chr03G007650	TRB
Macrophages 1	Carg_Chr08G010880	CLEC4C
Macrophages 1	Carg_Chr16G007590	Mrc1
Macrophages 1	Carg_Chr17G010310	Clec10a
Macrophages 1	Carg_Chr03G003110	CD209
Macrophages1	Carg_Chr02G011730	Kcnc1
Macrophages 2	Carg_Chr03G001570	RUNX3
Macrophages 2	Carg_Chr12G006740	SPIC
Macrophages 2	Carg_Chr14G005380	csf1r1
Macrophages 2	Carg_Chr15G006450	MPEG1
Macrophages 2	Carg_Chr19G001910	mpp1
B cells	Carg_Chr08G006420	Pax5
B cells	Carg_Chr09G008040	Cd79a
B cells	Carg_Chr09G009200	Hmcn2
B cells	Carg_Chr10G000670	Pou2af1
B cells	Carg_Chr08G001930	CD22
Neutrophils	Carg_Chr01G002710	COLEC12
Neutrophils	Carg_Chr08G000430	SLC8A1
Neutrophils	Carg_Chr08G011960	Clec6a
Neutrophils	Carg_Chr12G005510	MMP9
Neutrophils	Carg_Chr18G001450	Ncf1

NK cells	Carg_Chr17G005410	Bcr
NK cells	Carg_Chr21G002520	ple3
NK cells	Carg_Chr22G002840	FLT3
NK cells	Carg_Chr23G001610	eomes
NK cells	Carg_Chr23G005290	ITGAM
Epithelial cells 1	Carg_Chr01G018640	Cdh1
Epithelial cells 1	Carg_Chr06G005000	GPA33
Epithelial cells 1	Carg_Chr11G001460	TP63
Epithelial cells 1	Carg_Chr14G000690	Fat2
Epithelial cells 1	Carg_Chr18G004790	POF1B
Epithelial cells 2	Carg_Chr07G000850	LAMB3
Epithelial cells 2	Carg_Chr15G008090	ITGA2
Epithelial cells 2	Carg_Chr16G010500	LAMC2
Epithelial cells 2	Carg_Chr21G006760	FERMT1
Epithelial cells 2	Carg_Chr24G002800	COL14A1
Epithelial cells 3	Carg_Chr04G003440	EHF
Epithelial cells 3	Carg_Chr06G000640	CUZD1
Epithelial cells 3	Carg_Chr07G003480	krt8
Epithelial cells 3	Carg_Chr08G009560	DMBT1
Epithelial cells 3	Carg_Chr10G008960	CLDN4
Fibroblasts 1	Carg_Chr01G015510	THBS2
Fibroblasts 1	Carg_Chr03G002630	THBS1
Fibroblasts 1	Carg_Chr13G006660	Enox1
Fibroblasts 1	Carg_Chr20G002500	HTR4
Fibroblasts 1	Carg_Chr20G003560	LOX
Fibroblasts 2	Carg_Chr10G002880	Capn1
Fibroblasts 2	Carg_Chr11G002190	pdgfra
Fibroblasts 2	Carg_Chr11G002640	PLPP3
Fibroblasts 2	Carg_Chr11G010140	Prrx1
Fibroblasts 2	Carg_Chr21G002460	scara5
Endothelial cells	Carg_Chr05G004740	Ptprb
Endothelial cells	Carg_Chr07G002470	FGD5
Endothelial cells	Carg_Chr08G011380	FCRL2
Endothelial cells	Carg_Chr09G006990	RASIP1
Endothelial cells	Carg_Chr20G006300	EGFL7
Mesangial cells	Carg_Chr04G007640	CALD1
Mesangial cells	Carg_Chr04G009620	TNS1
Mesangial cells	Carg_Chr04G010400	ZEB2
Mesangial cells	Carg_Chr17G006250	RBPMS
Mesangial cells	Carg_Chr07G008360	Lmod1
Adipocytes	Carg_Chr06G007540	MMP2
Adipocytes	Carg_Chr14G007050	RPL26
Adipocytes	Carg_Chr09G004660	OSBPL3
Adipocytes	Carg_Chr07G005160	RPS26

Adipocytes	Carg_Chr20G005780	RPL37
Goblet cells	Carg_Chr01G009680	agr2
Goblet cells	Carg_Chr04G006330	MUC5B
Goblet cells	Carg_Chr06G009590	MUC5AC
Goblet cells	Carg_Chr06G009600	Muc2
Goblet cells	Carg_Chr13G002600	slc12a8
Dendritic cells	Carg_Chr16G001080	IRF4
Dendritic cells	Carg_Chr16G007340	XCR1
Dendritic cells	Carg_Chr17G009660	HLA-DPA1
Dendritic cells	Carg_Chr17G009800	H2-Eb1
Dendritic cells	Carg_Chr20G003580	PSAP
Erythroid-like and erythroid precursor cells	Carg_Chr01G002180	Mr1
Erythroid-like and erythroid precursor cells	Carg_Chr02G007430	hba
Erythroid-like and erythroid precursor cells	Carg_Chr02G007440	hbb1
Erythroid-like and erythroid precursor cells	Carg_Chr15G005520	Ank1
Erythroid-like and erythroid precursor cells	Carg_Chr21G003490	RHAG
Epithelial stem cells 1	Carg_Chr01G004990	mef2d
Epithelial stem cells 1	Carg_Chr11G001810	CDKL5
Epithelial stem cells 1	Carg_Chr11G009200	Rgs4
Epithelial stem cells 1	Carg_Chr17G003950	SLC12A3
Epithelial stem cells 1	Carg_Chr21G005510	MYB
Epithelial stem cells 2	Carg_Chr01G014580	prom1a
Epithelial stem cells 2	Carg_Chr14G001270	ANXA5
Epithelial stem cells 2	Carg_Chr19G000210	ATP6V1A
Epithelial stem cells 2	Carg_Chr19G001010	aldoc
Epithelial stem cells 2	Carg_Chr23G005380	Ca4
smooth muscle cells	Carg_Chr01G015200	MLIP
smooth muscle cells	Carg_Chr11G005720	Obscn
smooth muscle cells	Carg_Chr11G005760	NEXN
smooth muscle cells	Carg_Chr13G006450	Ttn
smooth muscle cells	Carg_Chr17G004250	ACTN3

Table S2: Quality control information.

Sample	Bfn	Afn	Pct	Bfm UMI	Afm UMI	Bfm genes	Afm genes
Black	10429	9586	91.92%	554	530	446	428
White	7490	6824	91.11%	958	895.5	727.5	690

Note: Sample: Sample name. Black: normal *Channa argus*. White: albino *C. argus*. Bfn: The total number of cells detected before quality control filtration. Afn: The total number of cells retained after quality control filtration. Pct: The cell retention percentage, calculated as: Afn/Bfn. Bfm UMI: The median UMI per cell before filtration, reflecting the total number of transcripts captured in the cells. Afm UMI: The median UMI per cell after filtration. Bfm genes: The median number of genes detected per cell before filtration. Afm genes: The median number of genes detected per cell after filtration.

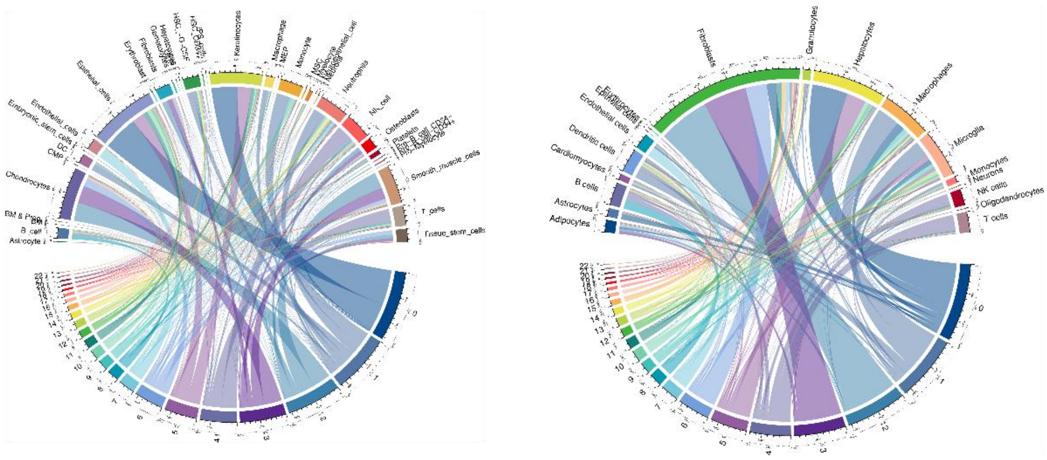

(A) (B)

Figure S1 Cell types inference corresponds to circos maps.

Note: (A) Cell annotation using singleR based on the marker genes database of human skin. (B) Cell annotation using singleR based on the marker genes database of mouse skin.
